# Docking and MM study of non-structural protein (NS5) of Japanese Encephalitis Virus (JEV) with some derivatives of adenosyl

**DOI:** 10.3389/fchem.2023.1258764

**Published:** 2023-11-27

**Authors:** Rakesh Kumar Tiwari, Vinayak Pandey, Harshita Srivastava, Ambrish Kumar Srivastava, Vishnudatt Pandey

**Affiliations:** Department of Physics, Deen Dayal Upadhyaya Gorakhpur University, Gorakhpur, Uttar Pradesh, India

**Keywords:** QM/MM, MD simulation, QM/MM-GBSA, JEV, S-adenosyl, binding energy, NS5

## Abstract

**Introduction:** The flavivirus NS5, a non-structural protein of Japanese Encephalitis Virus (JEV), a serious deadly human pathogen responsible for epidemics in South East Asia, consists of N-terminal methyl transferase (MTase) domain and RNA-dependent RNA polymerase (RdRp) is known for unique viral genome replication and cap formation activity. S-adenosyl executes a crucial function in these viral activities. S-adenosyl derivatives are chosen as potential binders with the MTase domain of NS5 based on MM and docking studies.

**Methods:** MM GBSA (Generalized Born Surface Area) simulation were performed to evaluate the binding energy, following the 100 nanosecond (ns) production MD simulation in the periodic boundary condition (PBC) for the selected docked ligands with NS5. Quasi-harmonic entropy of the ligands was also calculated with semi-empirical calculations at the PM3/PM6 level supporting docking and MM-GBSA results.

**Results and discussion:** The residue-wise decomposition energy reveals that the key hydrophobic residues Gly 81, Phe 133, and Ile 147 in the RdRp-MTase interface, indicate the biological relevance. These residues act as the key residue stabilizer, binding vigorously with S-Adenosyl derivatives in the vicinity of the interface between the MTase domain and RdRp. This paves the way for the other potential drug as an inhibitor for the enzymatic activity of the NS5.

## Introduction

The Flaviviridae family Japanese Encephalitis Virus (JEV), genus mosquito-borne, is a flavivirus hosting a positive-sense single-stranded RNA genome of 9,500–12,500 long nucleotides. It has spread rapidly across South East Asia particularly paddy-cultivated land including the northern part of India, Japan, and Nepal, and has become a serious deadly human pathogen owing to its link to the death of millions of children and adults by severe neurological diseases such as mental fever and Guillain-Barr syndrome (Bollati et al., 2010). Currently, no effective antiviral drug or vaccine is available to eradicate this menace (Lu and Gong, 2013; Lu and Gong, 2017). NS5 is the largest and most conserved non-structural protein of JEV plays a crucial role in viral genome replication and capping process and is considered a target with the different ligands (Malet et al., 2007). The high-resolution crystal structures are either available for MTase alone or RdRp.

The NS5 consists of an N-terminal S-Adenosyl-L-Methionine (SAM), known as SAMe. In the molecule, there was a SAM-dependent methyl transferase-(MTase) domain and the C-terminal RNA-dependent RNA polymerase (RdRp) domain that harbors the classic thumb, palm, and fingers domains present in all single sub-unit polymerase, and are central to the viral replication and capping (RC) with NS5 (Gu et al., 2007). The functions of NS5 are crucial as it harbors both the MTase domain bearing 5′ cap structure and RdRp for all RNA viruses which are responsible for inter-regulations and co-operativity and hence hypothesized as a prosperous site of drug activity (Kofler et al., 2006; Gu et al., 2007; Liefhebber et al., 2009; Bollati et al., 2010; Sztuba-Solinska et al., 2013). The S-Adenosyl, amino acid derivatives, normally synthesized in the body that may become depleted with sickness or age, is important as a potential ligand due to their activity in the liver and brain, especially antiviral, and is a major methyl donor in the synthesis of hormones, nucleic acids, proteins, and phospholipids, and catecholamines and other neurotransmitters (Bottiglieri, 2002; Krzystanek et al., 2011; Kotandeniya et al., 2018). SAMe (S-Adenosyl-Methionine) is required for the synthesis of norepinephrine, dopamine, and serotonin. SAMe facilitates glutathione usage, which improves the body’s antioxidant defense. It also helps to maintain acetylcholine levels which are necessary for cognitive function (Serra et al., 2008; Antoniv et al., 2017; Lanz et al., 2018). Similarly, S-Deaminosinfungin (SFN) commercially known as sinfungine (Lanz et al., 2018) belongs to the purine nucleosides. These are compounds comprising a purine base attached to a sugar. The proteins that adenosyl-ornithine target include RdmB, modification methylase TaqI, rRNA (adenine-N6-)-methyltransferase, and modification methylase RsrI, which was originally isolated from *Streptomyces*. Sinefungin proved useful as a non-selective inhibitor of SET domain-containing methyl transferases in the study of epigenetic regulations (Chan and Lithgow, 2008; Grove et al., 2011; Stubbe, 2011; Davis et al., 2017; Kim et al., 2017; LaMattina et al., 2017; Pinotsis and Waksman, 2017; Uppal et al., 2017; Zhang et al., 2017). S-adenosyl-3-thiopropylamine (SAT) is indicated as a potential inhibitor based on the S-Adenosyl homocysteine SAH scaffold (Se? kut? et al., 2011). Molecular S-adenosyl methionine (SAM) is used as a drug in Europe for the treatment of depression, liver disorders, fibromyalgia, and osteoarthritis. (Chan and Lithgow, 2008; Grove et al., 2011; Stubbe, 2011; Davis et al., 2017; Pinotsis and Waksman, 2017). It has also been introduced into the United States market as a dietary supplement for the support of bone and joint health, as well as mood and emotional wellbeing. Mechanics (MM) simulations inclusive of molecular docking are widely used to explore the time evolution of conformational aspects and binding aspects of biomolecules (Wang et al., 2001; Lee et al., 2016; Jo et al., 2017). Also, the cast factor popularizes the technique as supplementary to other experimental spectroscopic techniques.

The MD simulation methodology provides detailed microscopic modeling at the atomic scale as a powerful technique widely used in the research area of physics, chemistry, and materials science. The technique was used as a natural time evolution for molecular systems and allowed for the prediction of static and dynamic properties directly from the underlying interactions. The dynamic simulation is concerned with time-dependent processes in the molecular systems for their structural, dynamic, and thermodynamic properties by solving numerically the equations of motion. So, the MD simulations provide information about the time dependence and fluctuations in both velocity and positions. Although the MD simulation provides an approximate result, they are completely under the control of the users for changing and removing some specific constraints. During the calculations, their role and influence can be examined. The application of MD simulation can be classified mainly into three types (Karplus and McCammon, 2002):

1) They were used mainly for conformational sampling and refinement or determination of results obtained from X-ray, NMR, and other experimental techniques.
2) It was used to describe the properties of equilibrated systems. In this technique, thermodynamic property, root mean square deviation, thermal fluctuation, the motion of the center of mass, and correlation factors are generally estimated. This is assumed as the second stage of MD applications.
3) The motion and evolution with the simulation time.

Recently, the MD simulation combined with density functional theory (DFT) (Srivastava and Misra, 2021) has been applied to study a variety of problems of biomolecular interests (Gupta et al., 2020; Bhattacharya et al., 2022; Biswal et al., 2023; Parth Sarthi et al., 2023) including finding the potential targets for coronavirus 2 (Panda et al., 2023) and inhibitors for SARS-CoV-2 (Abhik et al., 2022).

## Methodology

The initial coordinates of S-Adenosyl derivatives were taken from Pub Chem based on the already reported crystal structure of the protein-ligand complex (NS5-SAH) with (Compound CID: 439155) (Lu and Gong, 2013; Wang et al., 2017), and the coordinates of the NS5 protein were obtained from the RCSB protein data bank (Prlic et al., 2016) (PDB ID: 4K6M). The missing hydrogen and other residues were added using the LEAP module of the AMBER14 package (Salomon-Ferrer et al., 2013). The protein molecule was treated by AMBER ff14SB. The partial atomic charges and missing parameters for ligands were obtained from the RESP (Comell et al., 1993) charge fitting method at the B3LYP/6-31++G(2d,2p) (Becke, 1988; Lee et al., 1988) using the optimized geometry obtained by the Hartree-Fock (HF) methodology at HF/6-31G(d,p) level of theory with the Gaussian 09 program (Frisch et al., 2009). The selection of coordinates of S-Adenosyl derivatives was done by the best score flexible ligand docking procedure with grid-based scoring using DOCK6 (Allen et al., 2015). The parameters of SAM-dependent ligands were generated using the Antechamber Module of AMBER14 for GAFF2 parameters. During the docking procedure, all bonds within the ligand were kept rigid and were allowed to be flexible under the first-order approximation of molecular flexibility with the protein (Lang et al., 2009; Allen et al., 2015). Initially, force field scores were obtained by molecular mechanics interaction energies, consisting of van der Waals and electrostatic components (Kuntz et al., 1982). These actions are performed by constructing grids of 0.3 Å across the receptor molecule in a suitable rectangular box. After a proper parameterization of the protein and ligand, the system was solvated in a truncated octahedral box with a TIP3P (Jorgensen et al., 1983) water model and extended up to 10 A° from the protein surface. The resulting charge of the prepared model was neutralized with counter ions depending upon the total charge of the system (Ibraimova and Wade, 1998).

NS5 contains 905 residues including N-terminal 265 residues SAM dependent MTase domain. It is highly analogous to all other flavivirus MTase crystal structures. In its C-terminus RNA-dependent RNA polymerase (RdRp), the MTase is connected by a 10-residue linker (residues 266–275) as the sequence is highly variable and has unknown conformations. The conformations of these variable residues are modeled by using the best possible comparative protein modeling using spatial restraint methodology (Sali and Blundell, 1993). The complexes are minimized with 500 possible orientations. The best conformation of the docked complex has been chosen from all other possible conformations. In our study, out of them, the best four conformations of the docked complex were passed through a molecular dynamics (MD) study.

All the MD simulations were performed by the different units by sander, a module of AMBER–14 (Case et al., 2005) package. The modeled systems were first minimized into two steps to get minimum energy conformations (Braun et al., 2019). In the first step of minimization, the protein was constrained by position restraint with a force constant of 50 kcal/mol-Å^2,^ and in the second step of minimization a full minimization without restraint. All minimizations were carried out by using 5,000 steps of steepest descent (Mega, 2010). Thereafter, the system was gently heated to the targeted temperature from 0 K to 50 K using restraint force 50 kcal/mol with the SHAKE (Ryckaert et al., 1977) algorithm used to constrain H-atoms. A similar strategy was used to further increase the temperature of the system from 50 K to 300 K in six steps, i.e., the interval of 50 K/cycle; each cycle followed by 1 ns dynamic. The third step, a constant pressure of 1.0 atm in the NPT ensemble for 10 ns dynamics at 300 K and at constant pressure using Langevin thermostat (Lzaguirre et al., 2001) and Bresendsen barostat (Berendsen et al., 1984). Constant pressure was maintained with a relaxation time of 2ps and temperature was controlled with a collision frequency of 2 ps^−1^. The density of the system is calculated to be 0.739g/cc at the given temperature and pressure. Now, using a Coulombic potential grid of 1 Å distance to neutralize the overall charges of the system. A finite boundary condition is applied for each ligand with NS5 sequence including water with counter ions Na^+^, Zn^2+^, and SO_4_^2−^ by keeping homogeneity at 4 Å distance from the molecular surface by considering a suitable unit shell. Now, using a Coulombic potential grid of 1 Å distance to neutralize the overall charges of the system. This was followed by equilibration for approximately 10 ns for each system then after production simulation was carried out for 100 ns to each system (Case et al., 2005; Dubey et al., 2013) without any restraints for the study of the behavior of the system. A 10 Å cutoff was used with the Particle Mesh Ewald (PME) method (Mendieta-Moreno et al., 2014) to treat long-range electrostatic and nonbonded interactions. MM-GBSA calculations (PM3 and PM6) were initiated after examining the production 100 ns trajectory.

The results were analyzed using the CPPTRAJ, a module available in the AMBER–14 packages, and molecular graphics images were produced by using the CHIMERA package (Beccuti et al., 2014) and the online tool Chem Draw Direct 1.5 (Probst and Reymond, 2018). A similar strategy was adopted for each set of the complex. The AMBER–14 nomenclature has been used to define atoms.

### Binding energy calculations

The free energy of binding, ∆G_bind_, is given by

$$∆G_{\text{bind}}=G_{P+D}–\;\left(G_{P}+G_{D}\right)$$ (1)

Where G_P + D_, G_P_, and G_D_ are the free energies of the complex, receptor, and ligand, respectively. In the MM/PBSA approach, each free energy term in Eq. 1 is calculated as:

$$G=E_{\text{bound}}+E_{\text{vdW}}+E_{\text{ele}}+G_{\text{PB}}+G_{\text{SA}}–\;{\text{TS}}_{S}$$ (2)

Where E_bound_ is the contribution from the molecular mechanics bond energy, i.e., the sum of the bond, angle, and dihedral energies. E_vdW_ is the van der Waals energy contribution and E_ele_ is the electrostatic energy in free energy. Similarly, T represents the absolute temperature while Ss represents the solute entropy. However, we ignored this term in this study (Lzaguirre et al., 2001). G_PB_ and G_SA_ are polar and non-polar contributions to the solvation energy, respectively, which were calculated by using the AMBER–14 software.

## Result and discussion

**FIGURE 1:**
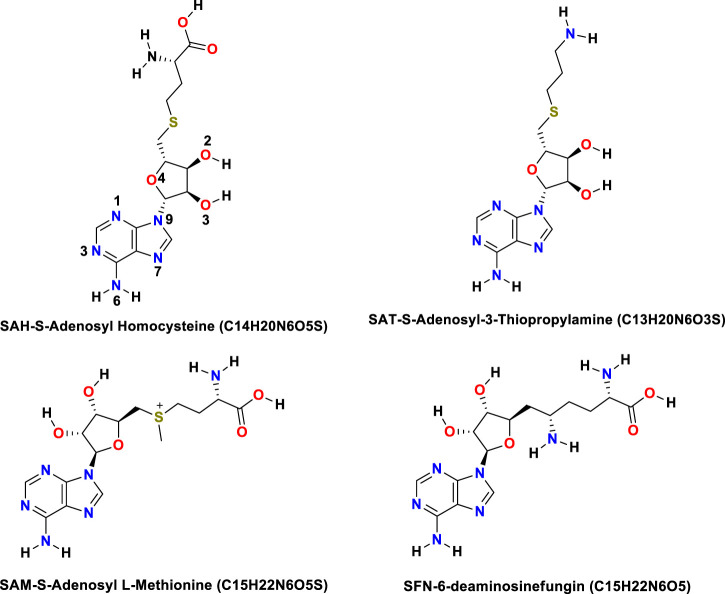
Structures of all the ligands (SAH, SAT, SAM, and SFN).

The binding energy of receptor-ligand was determined by the component analysis of MM GBSA calculation of many structures on the time interval of 1 ns of 100 ns dynamics of the ligand with 266 residues receptor (MTase). Here, we present molecular docking and molecular dynamics study of some derivatives of SAM shown in Table 1 and Figure 1.

**TABLE 1:** Docking analysis of different ligands in Kcal/mole.

Ligand	Grid score	vdw energy	Electrostatic energy	Internal energy (repulsive)
SAH	−64.604324	−42.840862	−21.763460	5.873916
SAM	−60.970791	−51.610973	−9.359818	12.808640
SAT	−53.453568	−42.693253	−10.760314	3.214480
SFN	−60.207264	−45.316769	−14.890495	7.897538

The Flavivirus NS5 represents a unique natural fusion of two important enzymes–MTase and RdRp, although MTase is common for viruses bearing a 5^′^ cap structure, and similarly, RdRp is required for all RNA viruses. However, the mechanism of inter-regulations and co-operativity between the two enzymes of NS5, i.e., MTase domain and RdRp domain remains elusive. The 10-residue linker not conserved in sequence would provide freedom for the sampling of other possible conformations to some extent, but larger-scale rearrangements may require additional flexibility at the MTase-RdRp junction. It has been argued that the N-terminal extension (residues 276–303) may play a role in such a process (Srivastava and Misra, 2021). This 28-residue region is sometimes covered as part of the MTase. The Grid score of any complex made by ligand and receptor is important to predict the potential binder for the receptor. We have selected a total of four residues among different S-Adenosyl derivatives which have comparable and satisfactory grid scores. The docking study successfully predicts the active site of the protein. The conformation of the active sides agrees well with the corresponding experimental study (Lu and Gong, 2013; Lu and Gong, 2017). The analysis indicates that the van der Waals component of energy plays a leading role over two other, electrostatic and internal energy components. The electrostatic component of the binding energy of SAM is the lowest among the ligands. Similarly, the internal repulsive component is the highest for this ligand. The study predicts that the active pocket of the receptor is made by approximately 33 residues of the MTase domain. These important amino acids are in the range from Val 55–Gly 58, Ile 78–Trp 87, Tyr 103–Glu 111, Val 130– Phe 133, and Phe 144–Glu 149.

### QM-GBSA analysis

**FIGURE 2:**
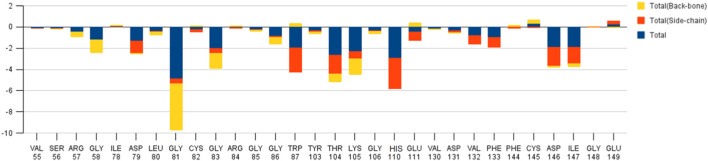
Residue-wise free energy analysis of SAH.

**FIGURE 3:**
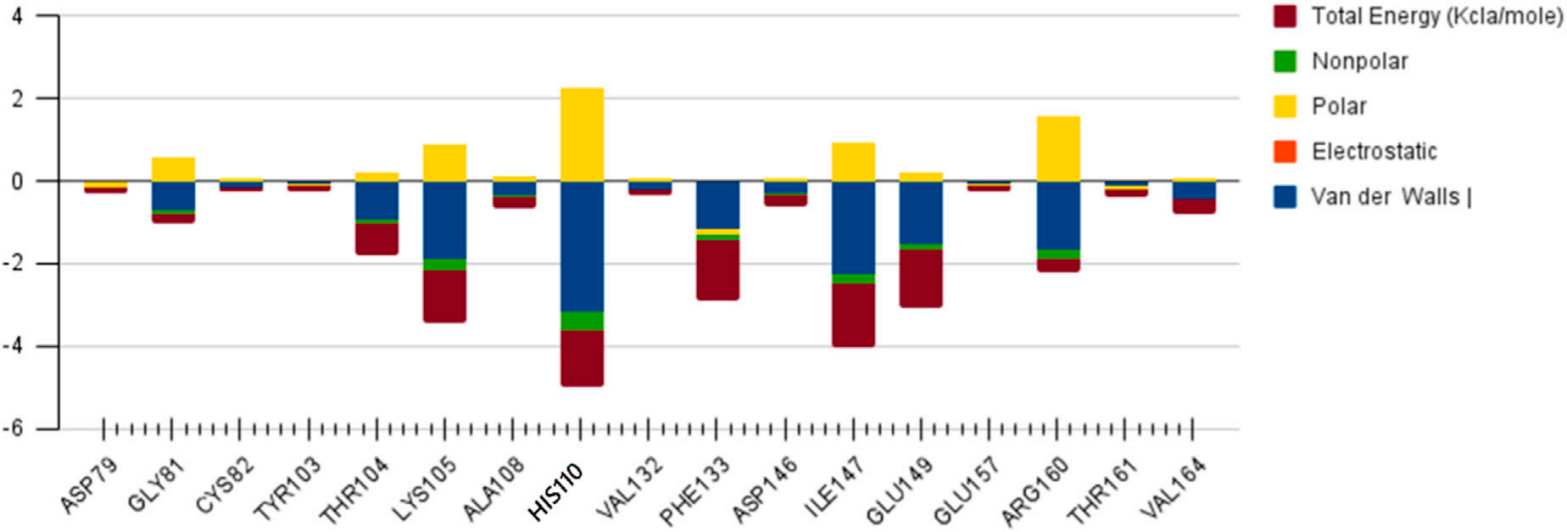
Residue-wise free energy analysis of SAM.

We again verify the results in light of molecular mechanics (MM) and Generalized Born Surface Area (GBSA) calculations. We took the initial coordinates of the docking simulation and performed the MM simulation, whose results are given in Figures 2, 3 for SAH and SAM, respectively. For MM-GBSA analysis, the free energy differences are calculated by combining the gas phase energy contributions that are independent of the chosen solvent model as well as solvation-free energy components (both polar and non-polar) calculated from an implicit solvent model for each species shown in Table 2. The ligands are here subjected to quantum mechanical calculations under PM3 and PM6 with a semi-empirical Hamiltonian for van der Waals and electrostatic energy separately. The different energy components are shown in Table 3. The contribution of translation, rotational and vibrational parts in entropies for different ligands are listed in Tables 4–7 for SAH, SAM, SAT and SFN, respectively.

**TABLE 2:** MM-PBSA free energy of different ligands with MTase domain of JEV in a.u.

Energy component	SAH	SAM	SAT	SFN
VDWAALS	−2209.6758	−2160.4899	−5.2031	−43.9828
E_EL_	−16778.1622	−16491.0957	−164.1186	−122.9479
E_GB_	−3770.6748	−3839.0018	−66.3304	134.6573
E_SURF_	89.8397	91.9507	3.7491	–5.8601
G_gas_	−18987.8380	−18651.5856	−169.3217	−166.9307
G_solve_	−3680.8351	−3747.0510	−62.5812	128.7972
TOTAL	−22668.6730	−22398.6367	−231.9029	−38.1335

**TABLE 3:** MM free energy of ligands with MTase domain of JEV in different simulations in Kcal/mole.

Ligand	MM-PBSA	MM-GBSA(PM3)	MM-GBSA(PM6)	Poisson–Boltzmann
SAH	−47.4685	−38.1335	−40.2117	−39.6529
SAM	−30.5444	−31.0538	−31.0580	−33.555
SAT	−35.6435	−30.9427	−30.9427	−24.1393
SFN	−40.9812	−30.3128	−30.3345	−16.2215

**TABLE 4:** Translational, Rotational, and Vibrational Entropies of SAH in Kcal/mole.

	Translational	Rotational	Vibrational	Total
Complex	16.9129	17.4698	10.1872	44.5698
Receptor	16.9018	17.465	10.1341	44.501
Ligand	13.0300	10.8383	5.5480	29.4164
∆*S*	−13.0190	−10.8336	−5.4949	−29.3475

**TABLE 5:** Translational, Rotational, and Vibrational Entropies of SAM in Kcal/mole.

	Translational	Rotational	Vibrational	Total
Complex	16.9725	17.5560	38.8018	73.3303
Receptor	16.9615	17.5491	38.7896	73.3005
Ligand	13.0620	10.6702	17.8917	41.6238
ΔS	−13.0509	10.6633	17.8795	−41.5940

**TABLE 6:** Translational, Rotational, and Vibrational Entropies of SAT in Kcal/mole.

	Translational	Rotational	Vibrational	Total
Complex	16.9117	17.4591	125.6422	160.0129
Receptor	16.9018	17.4549	125.5083	159.8650
Ligand	12.9221	10.6657	21.4307	45.0183
ΔS	−12.9123	−10.6613	−21.2969	−44.8704

**TABLE 7:** Translational, Rotational, and Vibrational Entropies of SFN in Kcal/mole.

	Translational	Rotational	Vibrational	Total
Complex	16.9126	17.4498	124.8792	159.2416
Receptor	16.9018	17.4459	124.7033	159.0511
Ligand	12.9850	10.8011	27.4817	51.2678
ΔS	−12.9743	−10.7972	−27.3058	−51.0773

The value of **Δ**S for SAH, SAM, SAT, and SFN is–29.4, −53.7, −44.8, and −51.1 kcal/mole respectively, which are consistent with the MM-BPSA result and satisfactory, near to our expectation.

### Quasi-harmonic estimation of free energy

Estimation of absolute conformational entropy is important because it allows a detailed understanding of the thermodynamic driving forces at the molecular level. This method calculates the thermodynamic conformational entropy of a bio-molecule during molecular dynamics simulation. Principal component analysis (the quasi-harmonic approximation) provides the first decomposition of the correlations in particle motion and entropy is calculated analytically as a sum of independent quantum harmonic oscillators.

**FIGURE 4:**
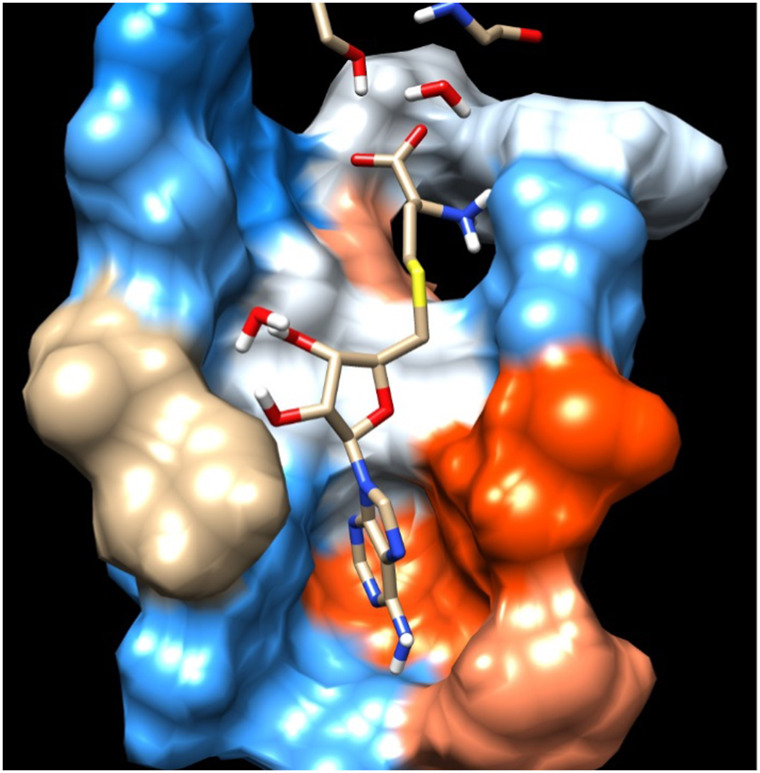
Residue interaction of SAH with Hydrophobic surface of MTase domain of JEV.

**FIGURE 5:**
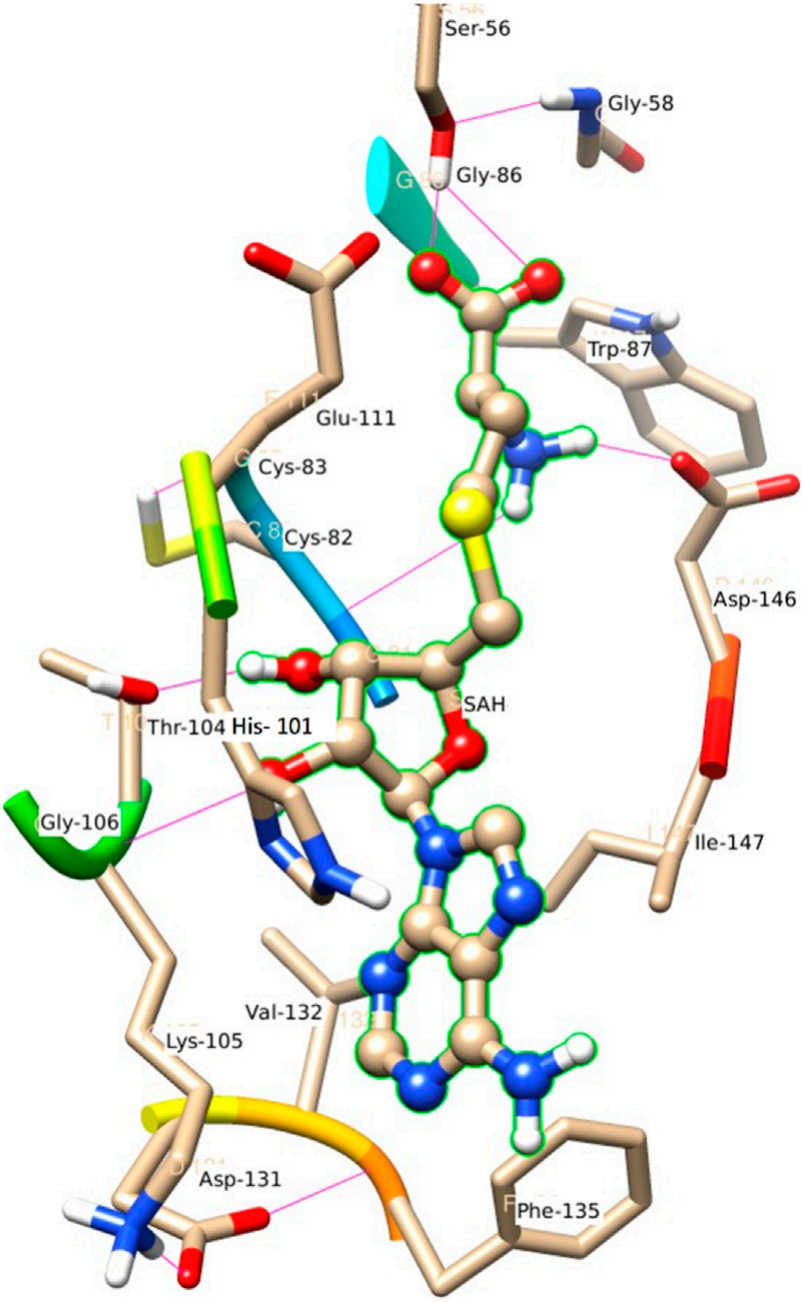
Another view of Residue interaction of SAH with MTase domain of JEV.

The decomposition energy per residue for the complex having SAH ligand shows that the residues His 110, Tpr 87, Thr 104, Asp 146, and Ile 147 (energy is in decreasing order in Kcal/mole) contribute larger as a side-chain composition. However, Gly 81, Lys 105, and Gly 58 are major backbone energy contributors. The backbone composition of Gly 81 (−4.4) was much higher in comparison to other residues. On the other hand, the contribution of energy through the side of the chain by the residue is marginal at about −0.4 kcal/mole. On the contrary, Gly 81 (−4.9) is identified as the most specific residue in binding energy terms along with the others in decreasing order of free energy such as His 110 (−2.9), Thr 104 (−2.6), Lys 105 (−2.3), Gly 83 (−1.97), Trp 87 (−1.95), Asp 146 (−1.9) and Ile 147 (−1.9). Interestingly, the residues Cys 82 and Arg 84 have significant contributions to side chain composition shown in the pictorial graph (Figures 4, 5), although, they do not have any significant backbone contribution. The electrostatic contributions of Asp 146, Gly 81, Asp 79, His 110, and Arg 57 are high and their energies are given in decreasing order against the van der Waals energy components. They produce a negative effect on the stability of the complex under consideration. Similarly, the polar solvation energy contributions of His 110, Glu 111, Phe 133, Ilu 14 7, and Gly 81 are significant as they are affecting the stabilization.

#### SAM

The role of Glu 149 (−0.925) and Glu 157 (−0.102) are significant and act as a major backbone contributor. On the other hand, all the 15 residues (Gly 81, Thr 104, Lys 105, Gly 106, Ala 108, His 110, Val 132, Phe 133, Asp 146, Ile 147, Gly 148, Glu 149, Arg 160, Thr 161 and Val 164) have significant side chain contributors in terms of free energy. The pictorial graph of SAH and SAM reveals that the role of Ile 147, Phe 133, Lys 105, and Thr 104 are important as they provide van der Walls interaction of MTase and polar interaction of water. It has been concluded that the free energy contribution of Ile 147 (−1.5), Phe 133 (−1.5), Glu 149 (−1.4), His 110 (−1.34) Lys 105 (−1.28) from the residue side, in which van der Waals component of energy play a crucial role in comparison to electrostatic term. The polar and non-polar solvation terms emerged as second and third major contributors in the free energy, respectively.

#### SAT

The residue Gly 148, Ile 147, and Asp 146 have been immersed as a major backbone energy contributor along with Lys 105, Leu 80, Cys 82, and Thr 104. Similarly, Ile 147, Val 132, and Phe 133 are major side-chain energy contributors. The residue-wise free energy analysis found 16 residues, in which Ile 147, Gly 148, Val 132, Lys 105, Thr 104, and Asp 146 are important. The polar contribution of residue to this molecule is significant for Lys 105 (−0.9) and Asp 146 (−0.8), although their electrostatic component is repulsive. The residues Ile 147 (−2.0), Val 132 (−1.1), and Phe 133 (−1.1) are significant for side-chain contributions.

#### SFN

The electrostatic energy of Asp 146 is dramatically higher (−16.5) than the other 20 residues which have a free energy value of more than 0.1 kcal/mole. The free energy contribution of the residues Asp 146 (−8.6), His 110 (−2.9), Ile 147 (−2.6), Thr 104 (−2.3), and Arg 57 (−1.2) provide a major role in binding energy terms. Out of which Asp 146, His 110, and Ile 147 provide a side-chain contribution with the decreasing order of energy, while Arg 57, Val 130, Lys 105, and Ile 147 support as backbone contributors.

The main residues of receptor (MTase domain) reported as the potential binders were Gly 58, Asp 79, Gly 81, Gly 83, Trp 87, Thr 104, Lys 105, His 110, Phe 133, Asp 146, Ile 147, Glu 149, and Arg 160. Among them, the significant binders, binding with S-Adenosyl-x ligand are Lys 105, His 110, and Ile 147 with a wider range of total free energy contribution. The hydrophobic active residues with significant van der Waals component of energy among the total energy Ala 108 (−0.32) Phe 133 (−1.14) Ile 147 (−2.248) Val 164 (−0.419) Kcal/mole pre dominates in respect of ligands and MTase domain active sites. Hydrophobic interactions are short-range interactions that play an important role in the ligand-receptor binding affinities. It arises due to enthalpic and entropic effects. The hydrophobic interaction arises between two non-polar residues and due to the interaction, water is displaced from the interacting surfaces of two hydrophobic residues. Due to the expulsion of water, the surface area of the hydrophobic residues decreases leading to the formation of the ordered arrangement of water molecules around the solute. It was measured in terms of the decrease in entropy roughly proportional to the non-polar surface area of the molecule enhancing the molecular stability.

### H-Bonding analysis

#### SAH

**FIGURE 6:**
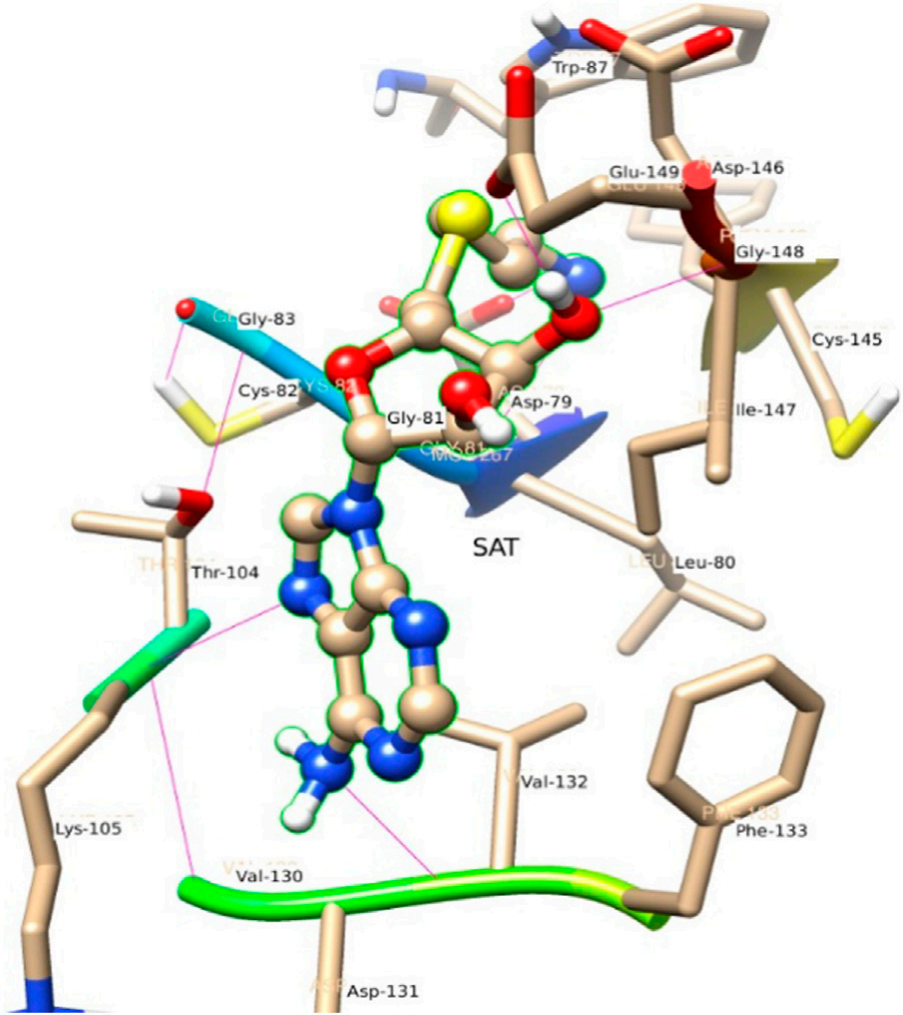
A view of residue interaction of SAT with MTase domain of JEV.

Figure 6 represents the H-bond with different residues of the MTase domain. The ligand makes H-bond with Ser 56, Asp 146, His 110, Thr 104, and Gly 81. The amide of the methyl group acts as a donor in the ligand and forms an H-bond with Asp 146 and Gly 81. Similarly, the amide group of Lys 105 acts as a donor and makes an H-bond with O_2_ of SAH. Ser 56 also has an important donor as it stabilizes the O atoms of SAH.

#### SAM

**FIGURE 7:**
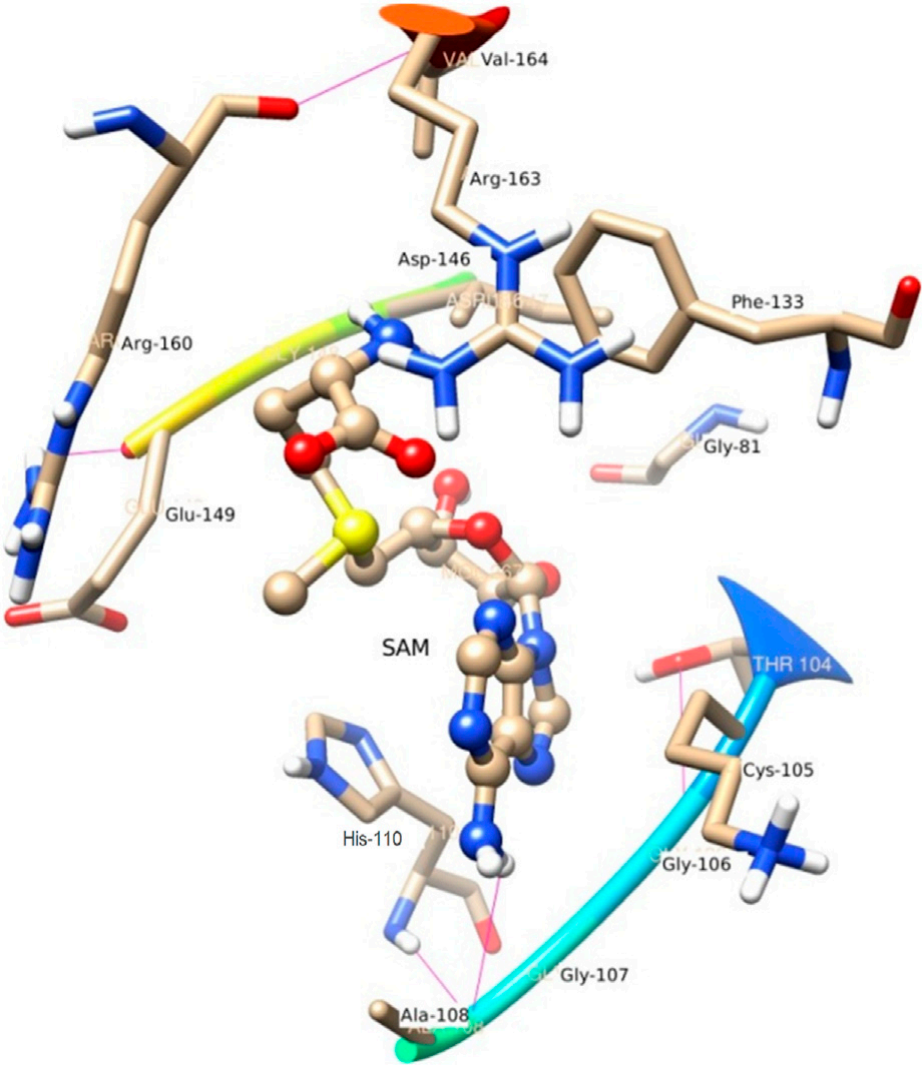
A view of Residue interaction of SAM with MTase domain of JEV.

N4 of SAM acts as a donor and makes H-bond with Gly 107. In the trajectory analysis number of water molecules as acceptor come out as a fraction, half of a water molecule, but the average number of H-bond as the acceptor comes out near to one water molecule. Figure 7 represents the conformation of the molecule inside the MTase domain. In the hydration analysis of the molecular dynamics trajectory, C9 of the furanose ring of the ligand appears as a powerful donor. It engaged itself as a donor to donate a proton to solute water molecules reported during the dynamics run by a 0.02 fraction of time. Similarly, N4, O5, C13, and O4 also act as a donor to create an H-bond to the water molecule of the solution at different snap-shot simulation periods. The H-bond formed by SAM is shown in Figure 7. Interestingly, the residues of MTase are more highly exposed than the other molecules such as water and ligands in the cavity to stabilize itself, which is also clear from its solvation-free energy. In the trajectory analysis, water molecules ranging from (10–35) have been found within the 3.5 Å shell of the ligand. An average of 20 H_2_O molecules are found with the first hydration shell. Similarly, 41 water molecules are found within 5 Å shells.

#### SAT

Figure 6 represents the conformation of the ligands inside the pocket of the MTase domain. H-bonds are represented by a dotted line. N1 of ligand makes H-bond with Asp 79, Liu 80 as a donor. N of Lys 105, makes H-bond with N3 of the ligand. Similarly, Val 132 acts as a donor and makes an H-bond with N4 of the ligand shown in Figure 6.

#### SFN

**FIGURE 8:**
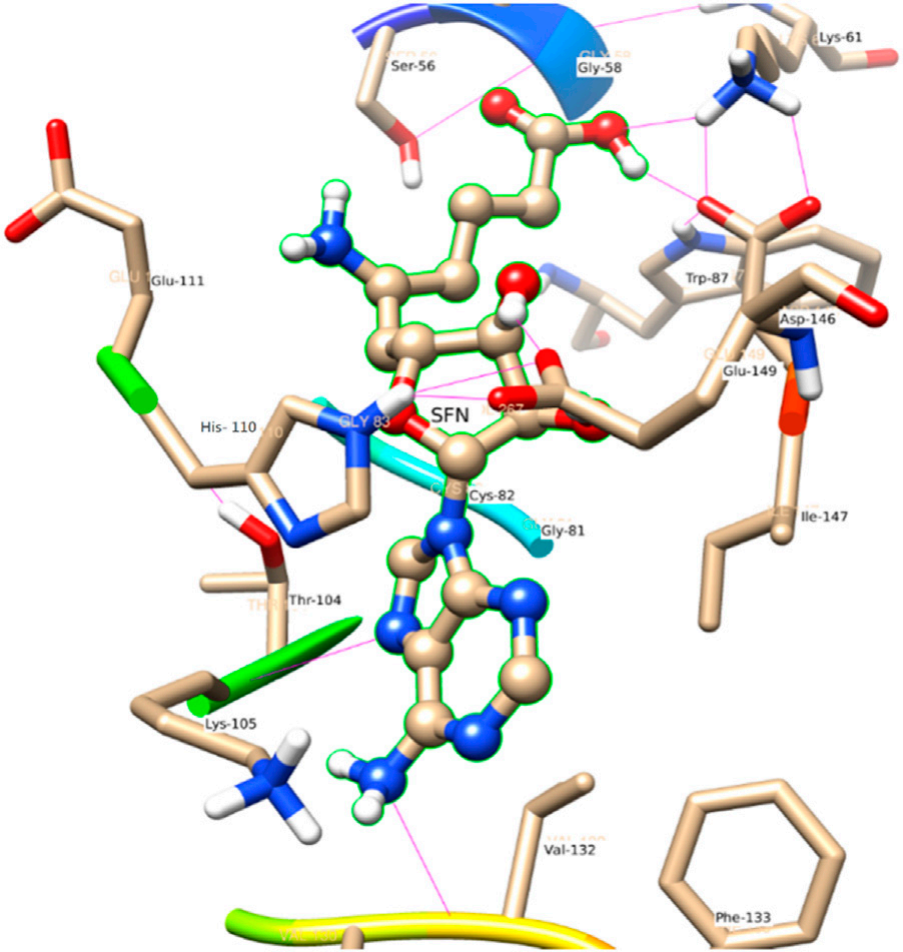
A view of residue interaction of SFN with MTase domain of JEV.

In comparison to the above three ligands such as SAH, SAM, and SAT, O2 of the ligand SFN acts as a donor and makes an H-bond with Glu 149. Whereas in the case of Asp 146, O2 as well as O4 of the ligand SFN form H-bond. Similarly, O5 forms an H-bond with Glu 149, Lys 61, and Lys 105 shown in Figure 8.

### The MTase domain

In the crystal structure, the MTase domain adopts a canonical SAM-dependent MTase fold with multiple helices flanking around a conserved 7-stranded βsheet. The conformation of the JEV MTase domain is largely consistent with the simulated MTase structures. MTase structures exhibit the highest similarity among all structures except the binding site (Grove et al., 2011; Tachikawa et al., 2018). The high degree of structural conservation also suggests that the MTase domain is quite rigid, not much affected by the presence of its natural fusion partner RdRp confirms our theoretical findings, i.e., the RMSD of heavy atoms of the simulated structure concerning crystal structure is within 3 Å. However, a slight variation is observed within the binding site of the MTase made by approximately 33 residues. Our simulation results find that there is a groove or cavity of approximately 1,365 Å^2^ area on the active side of the JEV. The activity of this pocket can be understood by the accommodation capacity of the grove as SFN has a larger dimension inside the groove and has an almost rod-like shape inside the groove in comparison to other ligands. Similarly, SFN is in a folded shape just to accommodate itself within the active region. The folding of SAM in comparison to the other three is important due to the presence of the methyl group in the backbone. However, we observed a little distortion at the active surface of JEV and showed slight variation in the presence of different residues. Similarly, the shape of SAH and SAT are also not identical but they show reasonable bindings with MTase. The important torsional angle of these different ligands is shown in Table 8. Therefore, we may predict that the site made by these amino acids is important for ligand binding, and the ligands selected here and their analysis have the potential to be used as a future binder.

**TABLE 8:** Conformational analysis of different ligands in the pocket of MTase domain of JEV.

Torsional angles	SAH	SAM	SAT	SFN
C–N–C–O (Angles between adenosyl and purine plane)	49.243	152.3	−14.9	36.1
The angle between the purine plane and backbone	179.3	55.3-	139.2	
S–C1–C2–C3	159.935	59.8	−160.6	-
C3–C2–C1–C4	162	−82.4	-	-
C3–C2–C1–N1	-	-	171	-

### A comparison with Zika virus (ZIKV)

Zika virus (ZIKV), emerging as a global health threat. It is a mosquito-transmitted Flavivirus in the Flaviviride family, including Dengue (DENV), Yellow Fever Virus (YFV), West Nile Virus (WNF), Japanese Encephalitis Virus (JEV) and Tick-born Encephalitis Virus (TBEV) (Minor and Diamond, 2017). ZIKV is a small enveloped positive-sense single-stranded RNA virus (Lindenbach and Rice, 2003). Among the non-structural proteins, NS5 is the largest enzyme with 904 amino acids (Issur et al., 2009). It consists of two domains, an N-terminal methyltransferase (MTase) domain and RNA dependent RNA polymerase (RdRp) domain at the C terminal. The NS5 RdRp domain contributes to the viral RNA synthesis through a *de novo* initiation mechanism (Gody et al., 2017).

The crystal structure of NS5 of ZIKV revealed that the MTase domain consists of a SAM-binding pocket, cap-binding site, and positive RNA-binding site. Therefore, the suppression of MTase activity is a promising strategy for drug development or the development of anti-ZIKV agents. Both the SAM-binding pocket and cap-binding site of the MTase domain proved to be essential targets for the drug development of ZIKV (Duan et al., 2017). Several inhibitors targeting NS5 MTase SAM-dependent domain have been identified for drug development. A SAM analog called sinefungin has been shown to have an inhibitory effect on NS5 MTase of DENV and WNV with inhibitory concentration IC_50_ of approximately 0.63 μM and 14 μM respectively (Dong et al., 2008). Similarly, theaflavin is a natural compound as one of the components of tea, a polyphenol with various biological properties, such as anti-viral, anti-bacterial, anti-metabolic syndrome, and anti-tumor activities (Sahoo et al., 2016) is reported to bind the active residue (D146) to inactivate the ZIKV NS5 MTase site (Song et al., 2021).

## Conclusion

The study explores the effect of SAM-dependent ligands such as SAH, SAM, SAT, and SFN on the NS5 protein of JEV with MD simulations and free energy calculations. The thermodynamical analysis using MM GBSA showed that the ligands SAH, SAM, SAT, and SFN binding is mainly governed by hydrophobic and van der Waals interactions. The electrostatic interactions are relatively weak. Gly 81, His 110, and Thr 104 make strong H-bond with SAH. Lys 105, Phe 133, Ile 147, and Gly 149 make strong H-bond with SAM. Cys 82, Gly 83, Trp 87, Thr 104, and Glu 149 make strong H-bond with SAT. Similarly, Ser 54, Gly 81, Cys 82, and Thr 104 make strong H-bonds with SNF. In addition, Gly 81, His 110, Thr 104, and Lys 105 provide the most energetic interaction via its hydrophobic side chain and are diagnosed as the main contributor to the hydrophobic and van der Waals interactions. The residue-wise estimate of decomposition analysis finds some more amino-acids such as Val 55-Gly 58, Ile 78-Trp 87, Tyr 103-Glu 111, Val 130-Phe 133, and Phe 144-Glu149 are the key residues for complexes stabilization. These residues are in the close vicinity of the catalytic tetrad K 61, D 146, and E 218; therefore, they are potentially important for further drug discovery as well as can model a new drug that can interact more efficiently and must be a better binder. Also, the pattern of intra-molecular interaction between the MTase domain and RdRp was not available so far providing a great opportunity to study a full-length crystal structure of NS5 and proceed with the simulation to resolve the mystery of the active residues in the interface between MTase domain and RdRp.

## Data Availability

The original contributions presented in the study are included in the article/Supplementary Material, further inquiries can be directed to the corresponding author.
